# Wild‐type p53 enhances endothelial barrier function by mediating RAC1 signalling and RhoA inhibition

**DOI:** 10.1111/jcmm.13460

**Published:** 2018-01-24

**Authors:** Nektarios Barabutis, Christiana Dimitropoulou, Betsy Gregory, John D. Catravas

**Affiliations:** ^1^ Frank Reidy Research Center for Bioelectrics Old Dominion University Norfolk VA USA; ^2^ School of Medical Diagnostic & Translational Sciences College of Health Sciences Old Dominion University Norfolk VA USA

**Keywords:** P53, inflammation, barrier function

## Abstract

Inflammation is the major cause of endothelial barrier hyper‐permeability, associated with acute lung injury and acute respiratory distress syndrome. This study reports that p53 “orchestrates” the defence of vascular endothelium against LPS, by mediating the opposing actions of Rac1 and RhoA in pulmonary tissues. Human lung microvascular endothelial cells treated with HSP90 inhibitors activated both Rac1‐ and P21‐activated kinase, which is an essential element of vascular barrier function. 17AAG increased the phosphorylation of both LIMK and cofilin, in contrast to LPS which counteracted those effects. Mouse lung microvascular endothelial cells exposed to LPS exhibited decreased expression of phospho‐cofilin. 17AAG treatment resulted in reduced levels of active cofilin. Silencing of cofilin pyridoxal phosphate phosphatase (PDXP) blocked the LPS‐induced hyper‐permeability, and P53 inhibition reversed the 17AAG‐induced PDXP down‐regulation. P190RHOGAP suppression enhanced the LPS‐triggered barrier dysfunction in endothelial monolayers. 17AAG treatment resulted in P190RHOGAP induction and blocked the LPS‐induced pMLC2 up‐regulation in wild‐type mice. Pulmonary endothelial cells from “super p53” mice, which carry additional p53‐tg alleles, exhibited a lower response to LPS than the controls. Collectively, our findings help elucidate the mechanisms by which p53 operates to enhance barrier function.

## Introduction

P53 is involved in the regulation of various intracellular cascades which orchestrate molecular responses to numerous environmental stimuli. It governs cellular fate, by promoting cell cycle arrest, apoptosis or senescence. This transcription factor was discovered 30 years ago as the cellular partner of simian virus 40 large T antigen. A decade later it became clear that it is a potent tumour suppressor, which is frequently mutated in humans tumours 1.

Apart from its role in cancer, P53 is strongly involved in the defence of vascular endothelium against inflammatory insults. Inflammation is a major cause of endothelial barrier dysfunction and hyper‐permeability, leading to acute lung injury (ALI) and acute respiratory distress syndrome (ARDS) 2. The development of new therapeutic strategies against these devastating pathologies has been slow, and mortality of patients suffering from ARDS remains around 40% 3.

We have recently demonstrated that the induction of p53, by either HSP90 inhibition (by 17AAG) or Nutlin, inhibits the inflammatory RhoA pathway 4 which leads to MLC2 phosphorylation and subsequent actin stress fibre formation 5. 17AAG induced p53 by suppressing the expression of MDM2 and MDM4, the two major p53 negative regulators. Moreover, HSP90 inhibition suppressed the LPS‐induced p53 and MDM2 phosphorylation, modifications that increase the rate of p53 proteasomal degradation 6. *In vitro* studies on the effect of p53 silencing on endothelial monolayer permeability have confirmed that p53 is an essential element for the maintenance of vascular barrier function 4.

This study aimed to further investigate the mechanisms which orchestrate the protective effects of p53 against vascular dysfunction, focusing on the role of the two major small GTPases which exert prominent antagonistic roles on endothelial barrier function, namely Rac1 and RhoA 7.

Pharmacologic or genetic activation of Rac1 results in vascular barrier enhancement. Rac1 induces p21‐activated kinase (PAK1) phosphorylation that leads to PAK1 autophosphorylation and activation. Activated PAK1 phosphorylates LIMK1/2, which, in turn, phosphorylates the actin‐severing protein cofilin at Ser3 and inactivates it 8, leading to barrier protection. Further, in this study, P53 inhibition reversed the 17AAG‐induced down‐regulation of the cofilin PDXP. Conversely, activation of RhoA by numerous inflammatory mediators, including LPS, activates ROCK1/2 which in turn phosphorylates myosin light‐chain kinase II leading to actomyosin contraction, actin stress fibre formation and disruption of endothelial barrier integrity 7. Control of RhoA activation is complex and includes P190RhoGAP, a well‐known inhibitor of RhoA 5.

Here, we demonstrate that p53 is a key mediator of Rac1 signalling and, at the same time, inhibits RhoA signalling by inducing P190RhoGAP activation. Additionally, these findings shed light on previous observations 9 about the importance of HSP90 inhibitors as pluripotent anti‐inflammatory agents and suggest that p53 may act as a major intracellular defender inflammation‐triggered vascular barrier abnormalities.

## Materials and methods

### Reagents

17‐Allyl‐amino‐demethoxy‐geldanamycin (17‐AAG) was obtained from the National Cancer Institute (Bethesda, MD, USA). AUY‐922 was purchased from Selleckchem (Houston, TX, USA). P53 siRNA (sc‐29435), PAK siRNA (sc‐29700), P190RHOGAP siRNA (sc‐44077), PDXP siRNA (sc‐61425), control siRNA (sc‐37007) and MDM2 antibody (sc‐965) were purchased from Santa Cruz Biotechnology (Santa Cruz, CA, USA). p53 (9282s), p‐myosin light‐chain 2, cofilin (3318), phospho‐cofilin (3311), PAK1 (2602), LIMK1 (3842) and phospho‐LIMK1 (3841) antibodies were obtained from Cell Signaling (Danvers, MA, USA). Β‐actin antibody (P8999) and CelyticM lysis reagent (C2978) were purchased from Sigma‐Aldrich (St Louis, MO, USA). Secondary mouse and rabbit antibodies were purchased from Licor (Lincoln, NE, USA). Oligofectamine (12252011), Pierce BCA protein assay and nitrocellulose membranes were obtained from Fisher Scientific (Pittsburgh, PA, USA). Ad‐p53‐GFP (1260) and ad‐GFP (1060) were obtained from Vector Biolabs (Malvern, PA USA).

### Animals

Seven‐ to 8‐week‐old male C57BL/6 mice from Jackson Laboratories were used in all experiments. Global transgenic p53 (“super p53”) was generated according to previously published procedures 10. Mice were maintained under pathogen‐free conditions in a 12:12‐hr light: dark cycle. All animal care and experimental procedures were approved by the Old Dominion University IACUC and were in line with the principles of humane animal care adopted by the American Physiological Society.

### Cell culture

In‐house harvested human lung microvascular endothelial cells were isolated and maintained in M199 media supplemented with 20% FBS and antibiotics/anti‐mycotics, as described previously 11. Mouse endothelial cells were grown in Lonza EGM‐2 medium (CC‐3202).

### Isolation of mouse pulmonary endothelial cells

Lungs derived from mice were perfused with PBS, dissected into small pieces and transferred to a gentleMACS C tube (130‐093‐237) which contained enzyme mix from the MACS Mouse Lung Dissociation kit (130‐095‐927). The cells were filtered through a 70 uM MACS Smartstrainer (130‐098‐462), and the pellet was processed with the MACS Debris Removal Solution (130‐109‐398). The cellular suspension was incubated with mouse MACS CD45 Microbeads (130‐052‐301), washed and resuspended in PEB buffer and processed on the autoMACS PRO using the DEPLETES program. The negative fraction from this was then incubated with mouse MACS CD31 microbeads (130‐097‐418) and processed on the autoMACS PRO using the POSSELS program. The resulting positive fraction was dual‐labelled with MACS CD45‐APC‐Vio770 (130‐110‐773) and MACS CD31‐PE (130‐110‐807). MACSQuant Analyzer 10 was employed to verify a pure CD31 positive fraction.

### Measurement of endothelial barrier function

The barrier function of endothelial cell monolayers was estimated by electric cell‐substrate impedance sensing (ECIS) as previously published 12, utilizing an ECIS model 1600R (Applied Biophysics, Troy, NY, USA). All the experiments were conducted on confluent cells which had reached a steady‐state resistance of at least 800 Ω.

### Rac1 activity assay

Rac1 activation was detected by the Rac1 pull‐down activation assay (#BK035; Cytoskeleton, Denver, CO, USA). Briefly, 500 μg of cell lysates was incubated with GST‐Rhotekin‐RBD fusion protein and was coupled to glutathione resin. After precipitation, the complexes were washed with the lysis buffer, eluted in SDS‐PAGE sample buffer, immunoblotted and probed with Rac1 antibody. Aliquots were taken from supernatants prior to precipitation and were used to quantify total Rac1.

### Protein isolation, Western blot analysis and transfections

The procedure took place as previously described 4. The signal for the immunoreactive proteins was developed using the appropriate secondary antibody and was visualized in a LICOR Odyssey CLx imaging system. Transfections were performed according to a standard protocol 13.

### *In vivo* experiments

Stock solutions of *Escherichia Coli* LPS were prepared in saline. Mice received either vehicle (saline) or LPS (3000 unit/g of body weight, intratracheally) 24 h before receiving vehicle (10% DMSO in saline) or the Ηsp90 inhibitor, AUY922 (10 μg/g of body weight dissolved in 10% DMSO), intraperitoneally. Mice were killed 48 h later (*i.e*. 72 h after LPS) by cervical dislocation, and the lungs were flushed with 5 ml of ice‐cold PBS (5 mM EDTA), excised, dipped in saline, blotted dry, quickly snap‐frozen in liquid nitrogen, crushed to powder in a prechilled mortar and stored at −80^0^.

### Densitometry/statistical analysis

ImageJ software (National Institute of Health) was used to perform densitometry of immunoblots. All data are expressed as mean values ± S.E.M. (standard error of mean). A value of *P* < 0.05 was considered significant. GraphPad Prism 4 (version 4.03, Graph Pad Software, Inc., CA, USA) was used for data analysis. *n* represents the number of experimental repeats.

## Results

### Mouse lung microvascular endothelial cells (MLMVEC) from “super p53” mice overexpress p53 compared to wild type, but exhibit comparable sensitivity to LPS challenge, *in vitro*

**Figure 1:**
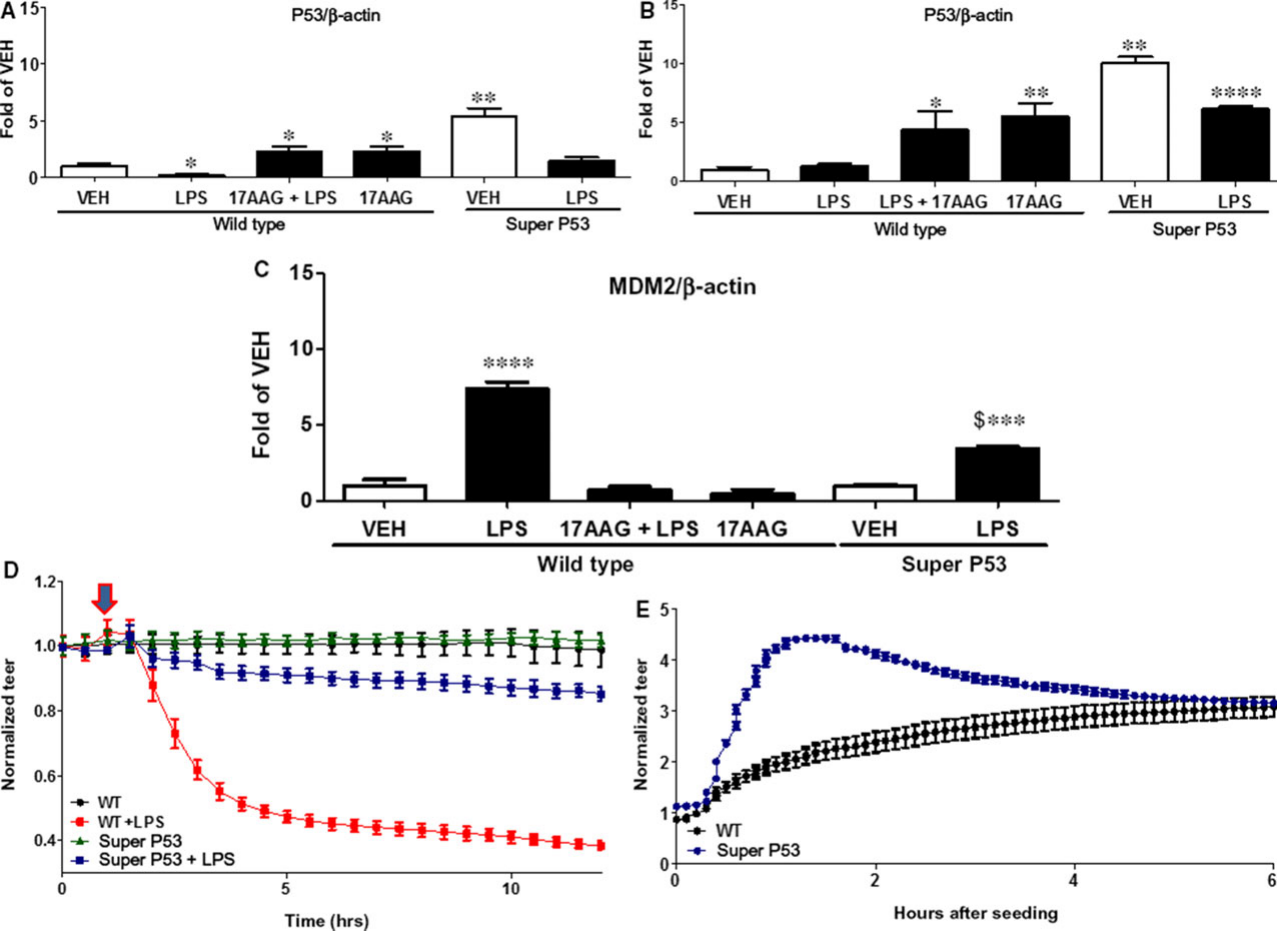
(**A**) Western blot analysis of p53 levels in mouse microvascular endothelial cells (MMVEC) isolated from lungs of wild‐type (WT) and super p53 mice. Cells were treated *in vitro* with LPS or vehicle and pre‐treated with 17AAG or vehicle (10% DMSO) for 16 h. Blot shown is representative of 3 experiments. Signal intensity was analysed by densitometry. Protein levels were normalized to β‐actin. *P <* *0.05 *versus* vehicle, **P <* *0.01 *versus* vehicle. Means ± S.E.M. (**B**) Western blot analysis of p53 levels in MMVEC derived from lungs of WT and super p53 mice 24 hrs after treatment with LPS or vehicle and pre‐treated for 16 h with 17AAG or vehicle (10% DMSO). Blot shown is representative of 3 experiments per group. Signal intensity was analysed by densitometry. Protein levels were normalized to β‐actin. *P <* *0.05 *versus* vehicle, **P <* *0.01 *versus* vehicle, *****P <* *0.0001 *versus* vehicle. Means ± S.E.M. (**C**) Western blot analysis of MDM2 levels in MMVEC isolated from lungs of WT and super p53 mice. The endothelial cells were treated *in vitro* with LPS or vehicle and pre‐treated for 16 hrs with 17AAG or vehicle (10% DMSO). Blot shown is representative of 3 experiments. Signal intensity was analysed by densitometry. Protein levels were normalized to β‐actin. *P <* *0.05 *versus* vehicle, **P <* *0.01 *versus* vehicle. Means ± S.E.M. (**D**) LPS was added to the media of MMVEC derived from the lungs of WT or super p53 mice. A gradual increase in endothelial permeability (reduced TEER) was observed in both LPS‐treated groups. However, WT cells were much more susceptible to LPS than those derived from super p53 mice. *n* = 4 per group. Means ± S.E. (**E**) Equal numbers of MMVEC derived from WT or super p53 mice were seeded on ECIS golden plated arrays and were allowed to form confluent monolayers. Cells derived from super p53 mice demonstrated an increased potential to reach confluence compared to WT cells. *n* = 4 per group. Means ± S.E.

MLMVEC isolated from wild‐type mice were treated *in vitro* for 16 h with either 0.1% DMSO (vehicle) or 17AAG before a 1‐hr treatment with PBS (vehicle) or LPS (10 EU/ml). The cells from the super p53 mice were only exposed to vehicle or LPS (10 EU/ml). MLMVEC from super 53 mice expressed significantly higher p53 levels. LPS reduced the expression of p53, and this was reversed in wild‐type cells treated with the HSP90 inhibitor, 17AAG. MLMVEC from super p53 mice exhibited a comparable decrease in p53 expression (Fig. 1A).

### *In vivo* treatment with LPS reduces MLMVEC p53 expression and induces MDM2 expression

*Wild‐type* mice received either vehicle (10% DMSO) or the HSP90 inhibitor, 17AAG intraperitoneally 24 hrs after vehicle (PBS) or LPS (intratracheally; it). Super P53 mice received only vehicle (PBS) or LPS it. 72 hrs after LPS, the animals were killed and MLMVEC were isolated and analysed by Western blotting. In cells from wild‐type mice treated with 17AAG, p53 expression was increased (Fig. 1B) and expression of the p53 suppressor MDM2 was decreased (Fig. 1C), in the presence or absence of LPS. LPS exerted minimal effect on p53 expression but dramatically up‐regulated MDM2 expression. Compared to cells from wild‐type mice, cells from super p53 mice exhibited again higher levels of p53, but unaltered levels of MDM2. Furthermore, *in vivo* treatment with LPS caused a strong decrease in p53 expression, but a less pronounced increase in MDM2 levels (Fig. 1B and C).

### MLMVEC derived from super p53 mice resist the LPS‐induced decrease in barrier function

MLMVEC were seeded on gold electrode arrays and were exposed to vehicle (PBS) or LPS (10 EU/ml), and transendothelial electrical resistance (TEER) was monitored continuously for the indicated times. MLMVEC from super p53 mice were more resilient to LPS, compared to cells from wild‐type mice (Fig. 1D).

### MLMVEC derived from super p53 mice reach confluence sooner that cells from wild‐type mice

Equal numbers (50,000) of MLMVEC derived from super p53 and wild‐type mice were seeded on golden plated electrodes and were left to grow till confluence. Figure 1E demonstrates that cells expressing higher levels of p53 exhibited a greater proliferative/spreading ability compared to wild‐type MLMVEC.

### Activation of Rac1 in human lung microvascular cells (HLMVEC) treated with 17AAG or AUY922

**Figure 2:**
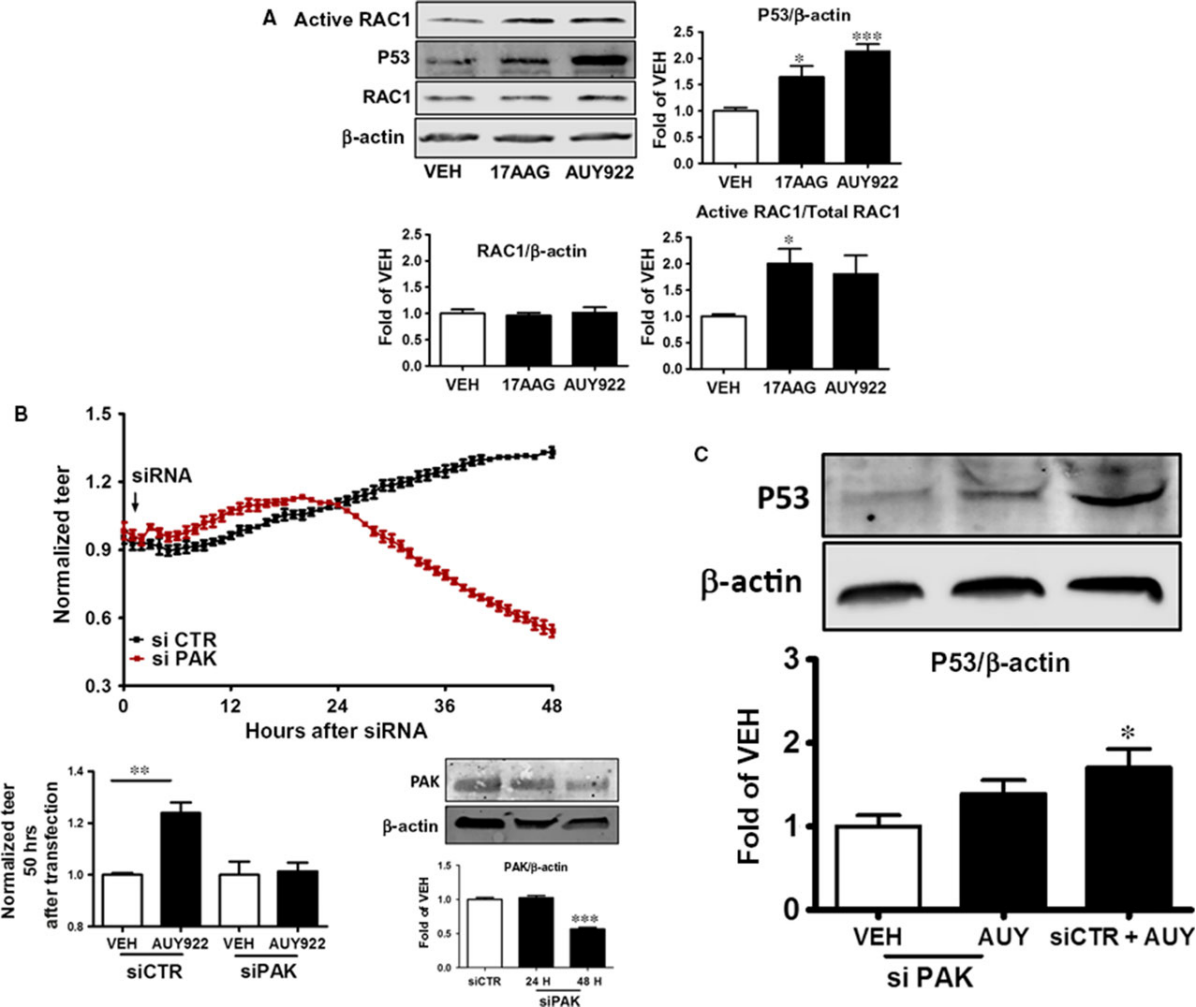
(**A**) Western blot analysis of active Rac1, P53, Rac1 and β‐actin after 8 hrs of treatment with either vehicle (DMSO; VEH) or 1 μΜ 17ΑΑG, or 1 μΜ ΑUY922 of human lung microvascular endothelial cells (HLMVEC). Blot is representative of 3 independent experiments. Signal intensity of active Rac1, P53, Rac1 was analysed by densitometry. Protein levels were normalized to Rac1 or β‐actin, as indicated. *P <* *0.05 *versus* vehicle, *****P <* *0.0001 *versus* vehicle. Means ± S.E. (**B**) HLMVEC were transfected with control (irrelevant) siRNA (si CTR) or PAK siRNA (si PAK) at t = 0. PAK siRNA‐treated cells exhibited reduced TEER values; *n* = 4 per group. Means ± S.E. In additional experiments, similarly treated HLMVEC were exposed to vehicle (0.1% DMS0) or AUY 922. Bars indicate normalized TEER values 50 hrs after transfection. *n* = 4 per group. ***P <* *0.001 *versus* vehicle‐treated cells; Means ± S.E. (lower left panel). Western blot analysis of PAK expression in HLMVEC 24 h an 48 h after siCTR or siPAK transfection. Blot shown is representative of 3 independent experiments. Signal intensity of PAK was analysed by densitometry. Protein levels were normalized to β‐actin.***P <* *0.001 *versus* control siRNA. Means ± S.E. (lower right panel). (**C**) HLMVEC were transfected with siCTR or siPAK and were consequently exposed to vehicle or AUY 922 for 16 hrs. Western blot analysis demonstrates p53 expression levels of transfected cells after 8 hrs of treatment with either vehicle or 17ΑΑG. Blot shown is representative of 3 independent experiments. Signal intensity of p53 was analysed by densitometry. Protein levels were normalized to β‐actin. *P <* *0.05 *versus* vehicle. Means ± S.E. (left panel).

HLMVEC were treated with either vehicle (0.1% DMSO), or the HSP90 inhibitors, 17AAG or AUY922 for 16 hrs. As shown in Figure 2A, both HSP90 inhibitors significantly induced Rac 1 activation and p53 expression.

### P21‐activated kinase (PAK) is essential for vascular barrier function and mediates the AUY922‐induced barrier strengthening

Rac1 activation is known to induce PAK. HLMVEC seeded on golden plated electrodes were transfected with siRNA which specifically targets PAK expression. The suppression of PAK, which is demonstrated in Figure 2B (lower right panel), resulted in compromised barrier function, as reflected in TEER values (upper panel, Fig. 2B). Moreover, PAK appears to be strongly involved in the AUY922‐triggered endothelial barrier enhancement, as silencing of PAK expression abolished the beneficial effect of HSP90 inhibition on HLMVEC barrier enhancement (Fig. 2B, lower left panel).

### PAK is essential for AUY922‐induced p53 expression

HLMVEC were exposed to siRNA specifically designed for the silencing of PAK gene expression (siPAK) as well as to an irrelevant siRNA (siCTR). 48 hrs after transfection, the cells were exposed to either vehicle (0.1% DMSO) or AUY922 (1 μΜ) for 16 hrs. In contrast to siCTR‐transfected cells, cells that expressed reduced PAK protein levels were unable to increase p53 expression in response to AUY922 (Fig. 2C). Thus, PAK appears to be downstream of AUY922 and p53 downstream of PAK.

### P53 induces the phosphorylation of LIMK

**Figure 3:**
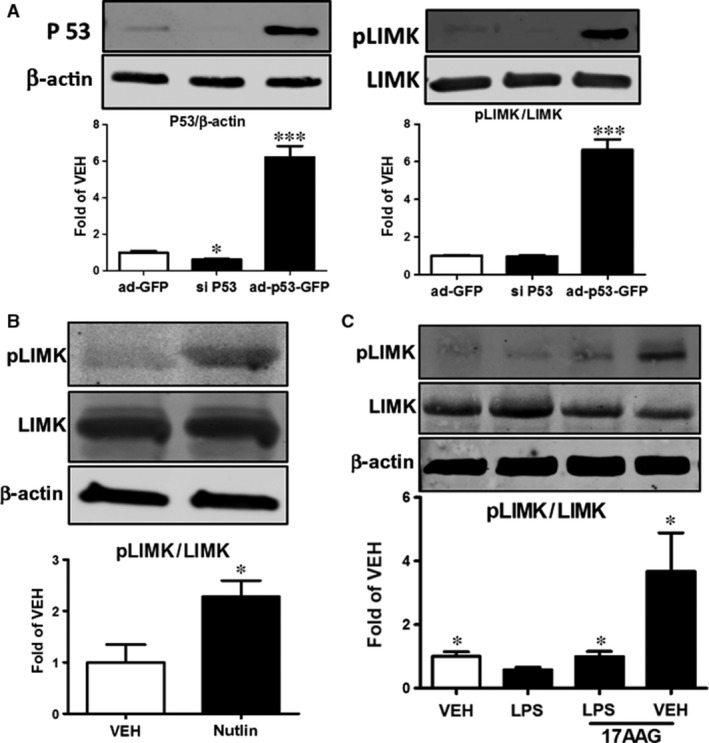
(**A**) Western blot analysis of p53 and β‐actin expression in HLMVEC after 48‐h treatment with ad‐GFP, siRNA for p53 (siP53), or ad‐p53‐GFP. Blot shown is representative of 3 independent experiments. Signal intensity of p53 was analysed by densitometry. Protein levels were normalized to β‐actin. *P <* *0.05 *versus* ad‐GFP, ***P <* *0.001 *versus* ad‐GFP. Means ± S.E. (left panel). Western blot analysis of pLIMK and LIMK levels in HLMVEC after 48‐h treatment with ad‐GFP, siP53 or ad‐p53‐GFP. Blot shown is representative of 3 independent experiments. Signal intensity of pLIMK was analysed by densitometry. Protein levels were normalized to LIMK. ***P <* *0.001 *versus* ad‐GFP. Means ± S.E. (right panel). (**B**) Western blot analysis of pLIMK, LIMK and β‐actin levels in HLMVEC after 48‐h treatment with vehicle (veh) (0.1% DMSO) or Nutlin. Blot shown is representative of 3 independent experiments. Signal intensity of pLIMK was analysed by densitometry. Protein levels were normalized to LIMK. *P <* *0.05 *versus* VEH. Means ± S.E. (**C**) Western blot analysis of pLIMK, LIMK and β‐actin levels in HLMVEC 1 hr after treatment with LPS or vehicle and pre‐treated for 16 h with 17AAG or vehicle. Blot shown is representative of 3 independent experiments. Signal intensity of pLIMK was analysed by densitometry. Protein levels were normalized to LIMK. *P <* *0.05 *versus* LPS. Means ± S.E.

**Figure 4:**
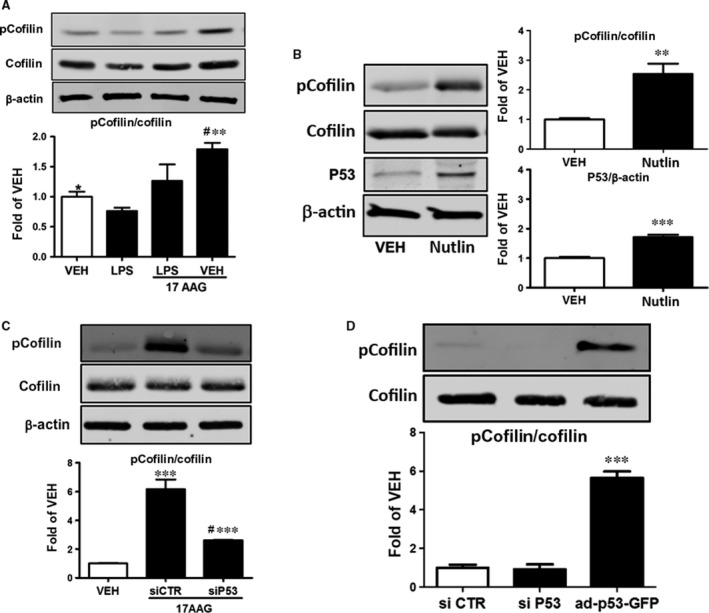
(**A**) Western blot analysis of pCofilin, cofilin and β‐actin levels in HLMVEC treated with LPS or vehicle and pre‐treated with 17AAG or vehicle (0.01% DMSO). Blot shown is representative of 3 independent experiments. Signal intensity of pCofilin was analysed by densitometry. Protein levels were normalized to cofilin. *P <* *0.05 *versus* LPS, **P <* *0.01 *versus* LPS, ^#^ *P *<* *0.05 *versus* vehicle. Means ± S.E. (**B**) Western blot analysis of pcofilin, cofilin, p53 and β‐actin levels in HLMVEC treated with vehicle or Nutlin. Blot shown is representative of 3 independent experiments. Signal intensity of pCofilin and p53 was analysed by densitometry. Protein levels were normalized to cofilin or β‐actin. *P <* *0.05 *versus* vehicle, ***P < 0.001 *versus* vehicle. Means ± S.E. (**C**) Western blot analysis of pcofilin, cofilin and β‐actin levels in HLMVEC transfected with either irrelevant siRNA (siCTR) or siRNA for p53 (siP53) and consequently treated with vehicle (0.01% DMSO) or 17AAG. The blot shown is representative of 3 independent experiments. Signal intensity of pCofilin was analysed by densitometry. Protein levels were normalized to cofilin. ***P <* *0.001 *versus* vehicle, ^#^ *P* < 0.05 *versus* siCTR. Means ± S.E. (**D**) Western blot analysis of pcofilin and cofilin in HLMVEC transfected with irrelevant siCTR, siP53 or ad‐p53‐GFP. Blot shown is representative of 3 independent experiments. Signal intensity of pCofilin was analysed by densitometry. Protein levels were normalized to cofilin. ***P <* *0.001 *versus* siCTR. Means ± S.E.

HLMVEC were transfected with ad‐GFP, si RNA which targets p53 gene expression (siP53), and ad‐p53‐GFP for 48 hrs. The efficiency of transfection is demonstrated in Figure 3A (left panel), where p53 was down‐regulated by siP53 and was dramatically induced by ad‐p53‐GFP. As shown on the right panel of Figure 3A, p53 overexpression resulted in the induction of phosphorylated (activated) LIMK (pLIMK). To confirm this finding, HLMVEC were treated for 48 hrs with either vehicle (0.1% DMSO) or Nutlin (10 μM), which we and others have shown to induce p53 expression (4 and Fig. 4B, lower right panel). Nutlin triggered the phosphorylation of LIMK, without affecting the levels of non‐phosphorylated LIMK (Fig. 3B).

### Effects of LPS and 17AAG on LIMK phosphorylation

HLMVEC were treated for 16 hrs with either vehicle (0.1% DMSO) or 17AAG (1 μM) before LPS or vehicle (PBS) treatment (1 hr). LPS suppressed LIMK phosphorylation and that effect was opposed by 17AAG, which, by itself, profoundly increased levels of pLIMK in HLMVEC.

### Effects of LPS and 17AAG on cofilin phosphorylation

HLMVEC were treated for 16 hrs with either vehicle (0.1% DMSO) or 17AAG (1 μM) before LPS or vehicle (PBS) treatment (1 hr). LPS suppressed cofilin phosphorylation and that effect was opposed by 17AAG (Fig. 4A). As shown by densitometric analysis of the blots, 17AAG treatment resulted in a nearly twofold increase in the deactivated cofilin (pCofilin) in HLMVEC.

### Nutlin‐induced p53 overexpression causes cofilin phosphorylation

HLMVEC were treated with 10 μM Nutlin or vehicle (0.1% DMSO) for 48 hrs. The Nutlin‐mediated p53 overexpression (Fig. 4B) increased the phosphorylated levels of cofilin (pCofilin), whereas total cofilin levels were not affected. We confirmed that this effect was due to p53 and not another irrelevant action of Nutlin by two approaches. HLMVEC were transfected with irrelevant siRNA (siCTR) or siRNA which targets p53 (siP53). 48 hrs after transfection, cells were treated with either vehicle (0.1% DMSO) or 17AAG (1 μΜ) for 16 hrs. Silencing of p53 expression blocked the 17AAG‐induced cofilin phosphorylation (pCofilin) (Fig. 4C). Additionally, HLMVEC were transfected with either scramble siRNA (siCTR), siRNA for p53 or ad‐p53‐GFP for 48 hrs. The induction of p53 expression resulted in profoundly increased phosphorylated levels of cofilin (Fig. 4D).

### Effect of LPS and HSP90 inhibition on phospho‐cofilin and P190RHOGAP levels in mouse lung microvascular endothelial cells (MLMVEC) of wild‐type and super p53 mice

**Figure 5:**
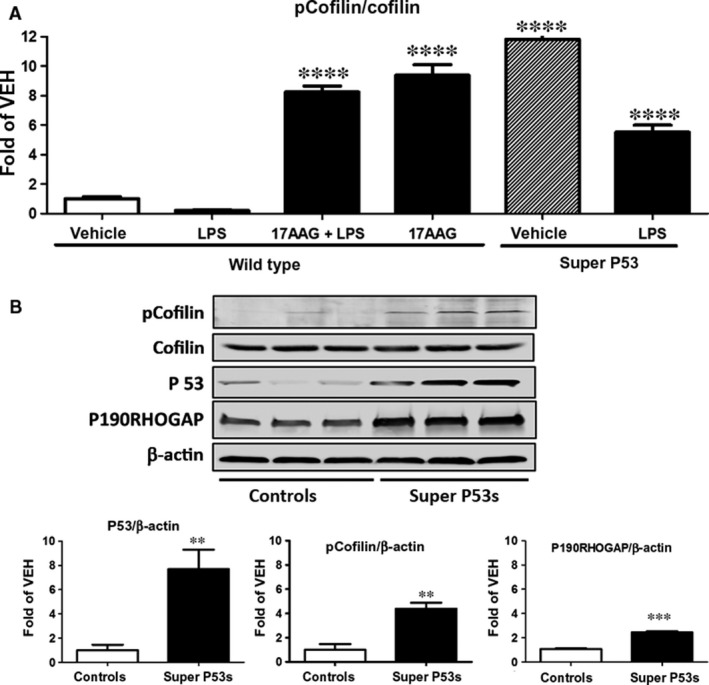
(**A**) Phospho‐cofilin expression levels in MMVEC isolated from lungs of wild‐type (WT) and super p53 mice. Cells were treated *in vitro* with LPS or vehicle and pre‐treated with 17AAG or vehicle (0.01% DMSO) for 16 h. Blot shown is representative of 3 experiments. Signal intensity was analysed by densitometry. Protein levels were normalized to cofilin. ****P <* *0.001 *versus* vehicle. Means ± S.E.M. (**B**) Western blot analysis of p cofilin, cofilin, p53, P190RHOGAP and β‐actin levels in lungs retrieved from WT and super p53 mice. Signal intensity was analysed by densitometry. Protein levels were normalized to cofilin or β‐actin. **P <* *0.01 vs controls (wild type). Means ± S.E.

MLMVEC isolated from wild‐type or super p53 mice were treated *in vitro* for 16 h with either 0.1% DMSO (vehicle) or 17AAG before a 1‐hr treatment with PBS (vehicle) or LPS (10 EU/ml). Cells exposed to LPS exhibited decreased expression of phospho‐cofilin (pCofilin) compared to the corresponding vehicle‐treated cells. Cells that were pre‐treated or treated with 17AAG expressed higher levels of pCofilin, compared to the corresponding controls (Fig. 5A). In additional experiments, **l**ung lysates from wild‐type and super p53 mice were analysed for the detection of pCofilin, cofilin and p53 protein levels. Results shown in Figure 5B indicate that super p53 mice express higher p53 expression levels in their lungs compared to wild‐type animals. Furthermore, these mutants express elevated levels of pCofilin and P190RHOGAP in their lungs compared to wild‐type mice.

### P53 inhibition reverses the 17AAG‐induced PDXP down‐regulation

**Figure 6:**
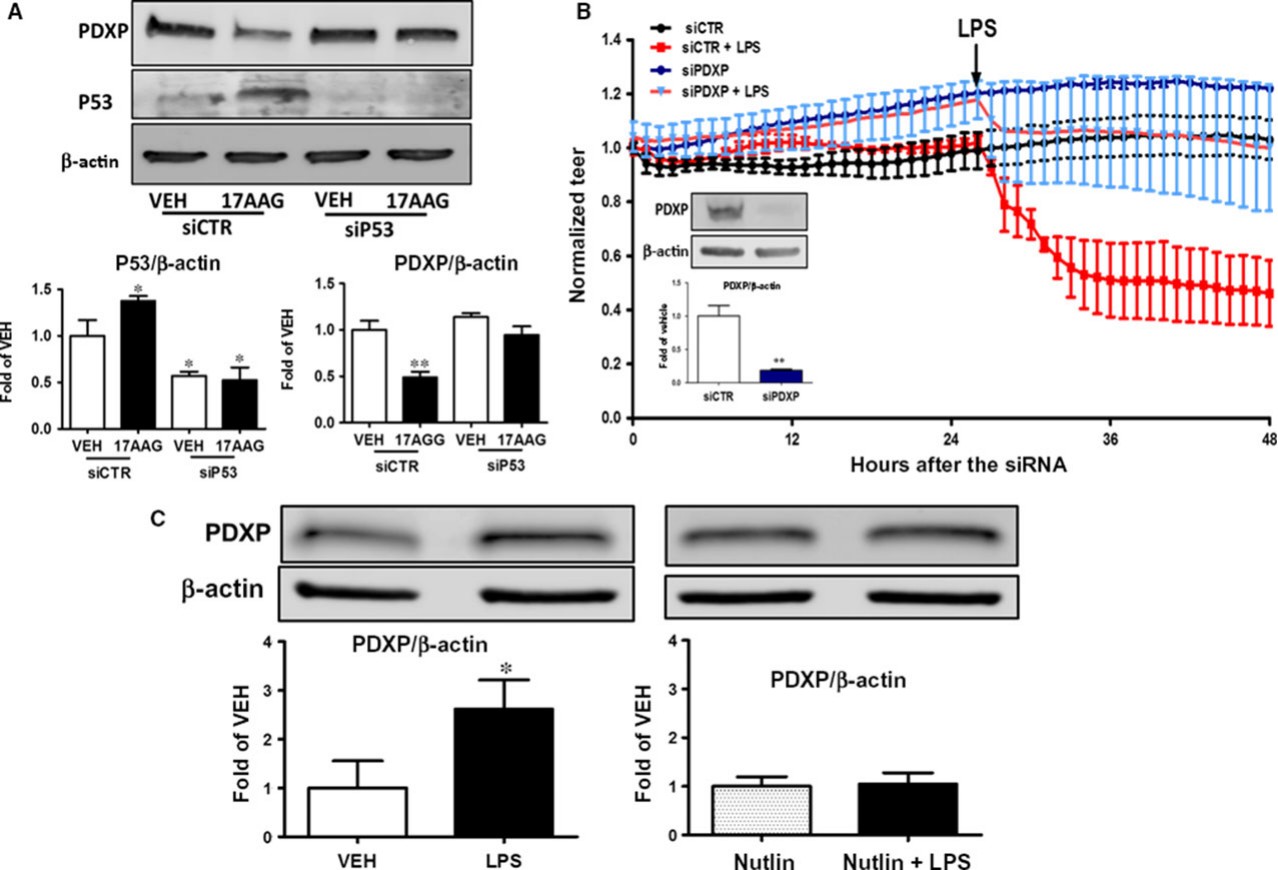
(**A**) Western blot analysis of PDXP, p53 and β‐actin protein expression in HLMVEC transfected with irrelevant siRNA (siCTR) or si RNA which targets p53 gene expression (siP53). Cells were then treated with vehicle (0.01% DMSO) or 17AAG. Blot shown is representative of 3 experiments. Signal intensity was analysed by densitometry. Protein levels of P53 and PDXP were normalized to β‐actin. *P <* *0.01 vs vehicle, **P <* *0.01 vs vehicle. Means ± S.E. (**B**) Cells were transfected with siCTR) or siPDXP and were then exposed to LPS. A gradual increase in endothelial permeability (reduced TEER) was observed in the LPS‐treated cells which were transfected with siCTR. Cells exposed to the si PDXP were not sensitive to LPS. *n* = 4 per group. Western blot analysis of PDXP and β‐actin protein expression in HLMVEC transfected with siCTR or siPDXP. Blot shown is representative of 3 experiments. Signal intensity of PDXP was analysed by densitometry. Protein levels were normalized to β‐actin. **P <* *0.01 vs siCTR. Means ± S.E. (**C**) Western blot analysis of PDXP and β‐actin protein expression in HLMVEC treated with LPS or vehicle (left), or pre‐treated with Nutlin prior to LPS or vehicle treatment (right). Blot shown is representative of 3 experiments. Signal intensity was analysed by densitometry. Protein levels of PDXP were normalized to β‐actin. *P <* *0.05 vs vehicle. Means ± S.E.

HLMVEC were transfected with irrelevant siRNA (siCTR) and p53 siRNA (siP53) for 48 hrs. Transfected cells were exposed to either vehicle (0.1% DMSO) or 17AAG (1 μM) for 16 hrs. Silencing of p53 blocked the 17AAG‐induced suppression of PDXP (Fig. 6A), an enzyme that catalyses the dephosphorylation of pCofilin 14.

### PDXP silencing blocks the LPS‐induced barrier dysfunction in HLMVEC

HLMVEC seeded on golden plated electrodes were transfected with siRNA which specifically targets PDXP expression (siPDXP) or irrelevant RNA (siCTR). In cells treated with siCTR, LPS caused an expected profound decrease in TER, indicative of endothelial barrier dysfunction. The silencing of the PDXP (shown in Fig. 6B, insert) completely blocked the LPS‐induced hyper‐permeability (Fig. 6B).

### Nutlin‐induced p53 expression counteracts the LPS‐triggered PDXP induction

**Figure 7:**
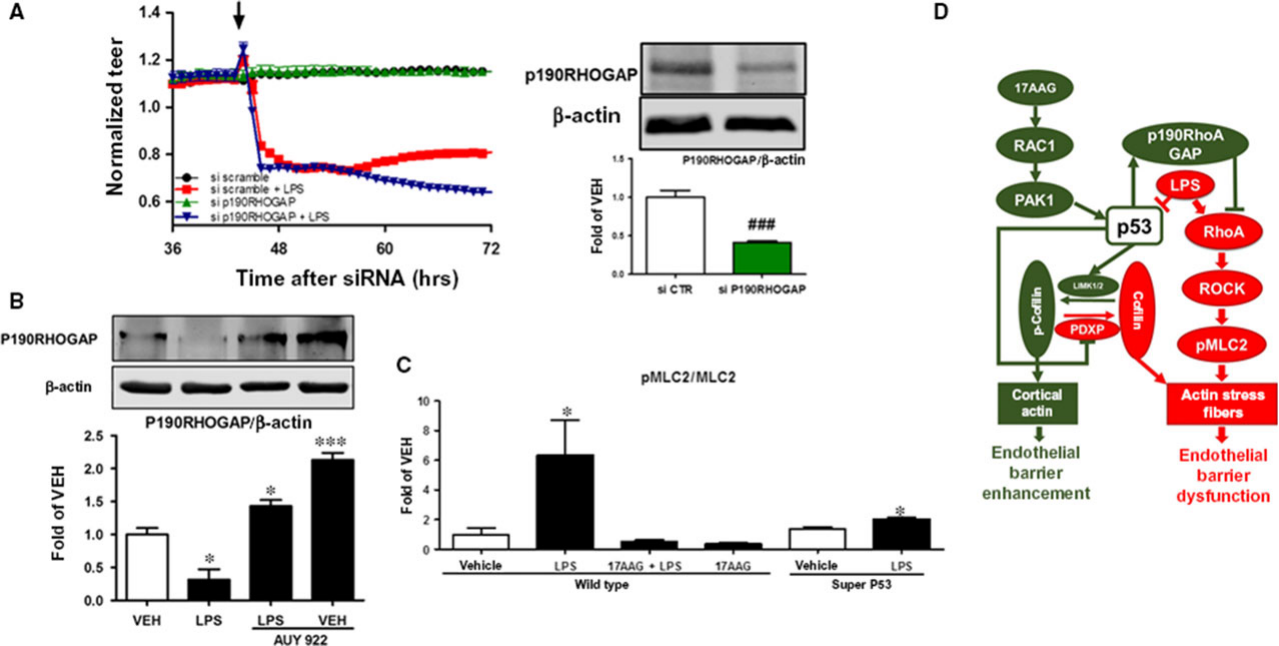
(**A**) Cells were transfected with irrelevant siRNA (siCTR) or siRNA targeting the p190RHOGAP gene expression and were then exposed to LPS. A gradual increase in endothelial permeability (reduced TEER) was observed in both LPS‐treated groups. However, cells exposed to si P190RHOGAP were more susceptible to LPS than those transfected with siCTR,* n* = 4 per group. Western blot analysis of P190RhoGAP and β‐actin protein expression in HLMVEC transfected with siCTR or si P190RHOGAP. Blot shown is representative of 3 experiments. Signal intensity of P190RHOGAP was analysed by densitometry. Protein levels were normalized to β‐actin. ^###^ *P *<* *0.001 vs siCTR. Means ± S.E. (right panel). (**B**) Western blot analysis of P190RHOGAP and β‐actin in HLMVEC treated with LPS or vehicle and pre‐treated with AUY922 (AUY) or vehicle (0.1% DMSO). Blot shown is representative of 3 independent experiments. Signal intensity of P190RHOGAP was analysed by densitometry. Protein levels were normalized to β‐actin. *P <* *0.05 *versus* LPS, ***P <* *0.001 *versus* LPS. Means ± S.E. (**C**) Phospho‐MLC2 expression levels in MMVEC isolated from the lungs of wild‐type and super p53 mice. Cells were treated *in vitro* with LPS or vehicle and pre‐treated with 17AAG or vehicle (0.01% DMSO) for 16 hrs. Blot shown is representative of 3 experiments. Signal intensity was analysed by densitometry. Protein levels were normalized to cofilin. *P <* *0.05 *versus* vehicle. Means ± S.E. (**D**) Schematic presentation of the proposed mechanism by which p53 regulates pulmonary barrier function by mediating Rac1 protective signalling and inhibiting barrier disruptive RhoA activation.

One‐hour treatment with LPS (1 EU/ml) greatly increased PDXP expression levels in HLMVEC (Fig. 7C, left panel). To investigate the role of p53 in this process, HLMVEC were exposed to Nutlin (10 μΜ) for 48 hrs prior to vehicle (PBS) or LPS (1 EU/ml) treatment. Nutlin completely suppressed the LPS‐induced PDXP induction (Fig. 6C, right panel).

### P190RHOGAP silencing enhances LPS‐induced barrier dysfunction

HLMVEC seeded on golden plated electrodes were transfected with siRNA which specifically targets P190RHOGAP expression (siP190RHOGAP) or irrelevant RNA (siCTR). The silencing of P190RHOGAP (Fig. 7A, right panel) further decreased TEER values and rendered the cells more sensitive to LPS (Fig. 7A, left panel).

### Effects of LPS and 17AAG on P190RHOGAP expression

HLMVEC were treated for 16 hrs with either vehicle (0.1% DMSO) or AUY922 (1 μM) before LPS (1 EU/ml) or vehicle (PBS) treatment (1 hr). LPS suppressed the P190RHOGAP expression and that this effect was strongly opposed by AUY922 treatment. Indeed, cells which were treated or pre‐treated with the HSP90 inhibitor exhibited higher levels of P190RHOGAP compared than vehicle‐treated cells.

### Effect of LPS on phospho‐MLC2 expression levels in mouse lung microvascular endothelial cells (MLMVEC) harvested from wild‐type and super p53 mice

MLMVEC isolated from wild‐type mice were treated *in vitro* for 16 h with either 0.1% DMSO (vehicle) or 17AAG before a 1‐hr treatment with PBS (vehicle) or LPS (10 EU/ml). Cells from super p53 mice were exposed to vehicle or LPS (10 EU/ml). All cells exposed to LPS exerted an increased expression of phospho‐MLC2 (pMLC2) compared to the corresponding vehicle‐treated cells (Fig. 7C), suggesting actin stress fibre formation. However, cells derived from super p53 mice exhibited a dramatically lower response to LPS than those from wild‐type mice. In wild‐type mice, 17AAG pre‐treatment completely blocked the LPS‐induced pMLC2 up‐regulation.

## Discussion

HSP90 regulates signalling cascades and protein trafficking by assisting the folding and maturation of proteins involved in the maintenance of cellular integrity and survival 12. Deregulation of this function in malignant cells contributes to the potentiation of metastatic growth. Thus, inhibition of HSP90 function by pharmaceutical agents was found to be a very attractive approach towards the development of new anti ‐ neoplastic agents 7.

There exists a strong association between cancer, inflammation and relevant organismal responses to bacterial toxins 15, 16, 17. Investigations on the association between the anti‐cancer activity of HSP90 inhibitors and their anti‐inflammatory properties have forged a strong body of evidence which not only supports the anti‐inflammatory activity of HSP90 inhibitors in the vasculature, but has specifically revealed a protective action against bacterial‐induced vascular hyper‐permeability 18. One of the many client proteins of HSP90 which are involved in cellular stability is the transcription factor and “guardian of the genome,” P53 19. In a series of independent studies 20, 21, p53 has been demonstrated to exert both anti‐cancer and anti‐inflammatory responses. Chronic inflammation promotes the development and progression of various epithelial tumours 22 Wild‐type p53 suppresses inflammation, and this effect is clearly linked to its tumour suppression function, as it was demonstrated in a mouse model of myeloid lineage‐specific p53 deletion or activation 23.

Remarkably, some bacteria have evolved to inhibit p53, a key component of the stress response machinery 24. Bacteria inhibit p53 through multiple mechanisms, including protein degradation, transcriptional inhibition and posttranslational modifications 24. Our laboratory has recently discovered the protective role of p53 on LPS‐induced vascular barrier dysfunction and that this novel property of P53 was associated with an LPS‐induced p53 suppression 4. This p53‐mediated protective effect is not strictly limited to LPS challenge. Want *et al*. 25 suggested that p53 protects mice against Listeria monocytogenes (LM) infection. p53 knockout (p53KO) mice were more susceptible to LM infection and showed significant impairments in LM eradication, presumably because of the abnormal production of the proinflammatory cytokines TNF‐α, IL‐6, IL‐12 and IL‐18. Silencing of p53 in RAW264.7 and HeLa cells resulted in increased invasion and intracellular survival of LM. These effects were inhibited in p53‐overexpressing RAW264.7 and HeLa cells. These results indicate that p53 serves as an important regulator of host innate immunity that protects against LM infection 25.

In the present study, we demonstrate that the cells which overexpress p53 (Fig. 1A and C) are less susceptible to LPS (Fig. 1D) and exhibit a greater proliferative capacity (Fig. 1D) compared to cells derived from wild‐type mice. In line with these observations, a recent study by Ticket *et al*. suggests that the suppression of p53 is associated with increased vascular permeability, which in turn causes aggressive tumour metastasis. The p53 suppression/inactivation was due to an AKT‐mediated MDM2 phosphorylation 26. We have previously demonstrated a similar MDM2 activation due to LPS treatment of HLMVEC 4.

We have previously described that HSP90 inhibition induces p53 expression by increasing the abundance of the HSP90/P53 complexes, by reducing p53 phosphorylation levels and by suppressing the expression of the P53 negative regulator, MDM2 4. We now report that HSP90 inhibition by two chemically distinct compounds, 17AAG and AUY922, resulted in p53 up‐regulation and activated Rac 1 induction (Fig. 2A). The former effect has been previously reported by our group and others 4, 27. In MCF7 breast cancer cells, activated Rac1/Cdc42 promotes ubiquitin‐mediated degradation of p53 to increase VEGF production 28. On the other hand, p53 prevents the initiating steps of filopodia formation, as its overexpression reduces protrusions 29. These protrusions are initiated by RhoA and opposed by Rac1 30. It was recently reported that the GTP‐bound RhoA induces Rac1 abundance 31 and that Rac1 inhibits RhoA 32. Thus, the crosstalk between these GTPases is regulated by a double‐negative feedback loop 31.

APE1 is the main apurinic/apyrimidinic endonuclease in eukaryotic cells, playing a central role in the DNA base excision repair pathway. In addition, it controls the intracellular redox state by inhibiting reactive oxygen species (ROS) production 33, 34. It was recently reported that APE1 overexpression decreases Rac1 35 activation, and it has already been established that p53 is a negative regulator of APE1 36. Thus, 17AAG‐ or AUY922‐induced P53 overexpression may suppress APE1 which in turn would up‐regulate Rac1 activation. Further, the reduced oxidative stress due to HSP90 inhibition 37 may also result in decreased APE1 levels, which in turn would induce Rac 1 activation.

The p21‐activated kinase (PAK) family of serine/threonine kinases is engaged in multiple cellular processes, including cytoskeletal reorganization 7. Rac1 induces the membrane translocation of downstream effectors and triggers their activation. Membrane‐bound Rac1‐GTP recruits p21‐activated kinases (PAKs) by binding to their Cdc42‐Rac interactive binding (CRIB) domain 31. In resting cells, PAK is localized in the cytoplasm as inactive dimers, with the regulatory domain shielding the kinase domain. Rac1 binding induces a subsequent activation of PAK, which in turn phosphorylate downstream substrates. PAK activity converts the local activation of Rho‐type GTPases into cell‐wide responses 38. The silencing of PAK in HLMVEC resulted in increased vascular permeability, an effect which is consistent with the strengthening role of PAK in barrier function (Fig. 2B). However, suppression of PAK protein expression partially prevented the AUY922‐induced p53 induction (Fig 2C). Rac1 closely collaborates with P53 to regulate major cellular functions such as cellular proliferation, transformation and motility. It has been reported that gain of Rac1 activity increases ROS production 39, which in turn has been found to induce P53 expression 17, 40. Further, it was suggested that Rac1 inhibition can elicit ERK1‐mediated P53 phosphorylation, which in turn results in p53 degradation 6. However, the exact mechanisms responsible for this regulation remain elusive 39.

LIMKs are downstream targets of PAK. Activated LIMK phosphorylates and inactivates the filamentous actin (F‐actin)‐severing protein cofilin. Spatially and temporally regulated cycles of cofilin inactivation and activation enable dynamic actin rearrangements required for cell motility 41. Croft et. al. have recently reported that in MCF7 cells, LIMKs are directly phosphorylated by P53 upon genotoxic stress and that LIMKs are a p53 target gene. Treatment with the antitumour drug doxorubicin reduces the activity of cofilin by increasing the expression of LIMK in a p53‐dependent manner 42. In line with these data, we now report that a similar regulation occurs in HLMVEC, as the manipulation of p53 expression levels by siRNA or ad‐p53 revealed a positive regulation between p53 and LIMK (Fig. 3A). Further, both Nutlin‐ and 17AAG‐induced p53 overexpression cause LIMK phosphorylation (Fig. 3B).

As cofilin is the downstream target of LIMK, we also tested whether p53 and HSP90 inhibitors affect cofilin. Figure 4A and B demonstrates that both P53 inducers deactivate cofilin by phosphorylation. Further, “silencing” of p53 by siRNA results in the suppression of 17AAG‐induced cofilin phosphorylation (Fig. 4C). P53 has been linked to actin polymerization 14. It was revealed that actin polymerization, which serves as a factor participating in the process of orchestrating p53 function in response to DNA damage, leads to the destabilization of p53 43. Binding of p53 to actin filaments is calcium dependent, and the interaction between these two entities is enhanced by DNA damage 44. Further, p53 interacts with G‐actin, shows perinuclear colocalization in response to genotoxic stress and translocates to the nucleus 45.

Mice lung microvascular endothelial cells isolated from wild‐type and super p53 mice were responsive to 17AAG and LPS treatment *in vitro*. Mice treated with the HSP90 inhibitor exerted increased levels of phospho‐cofilin, even when treated with LPS prior the 17AAG treatment (Fig. 5A). The induction of cofilin phosphorylation by agents that overexpress p53 did not occur only during *in vitro* experimentations. Super p53 mice that over express p53 10 also exhibit increased phosphorylated levels of cofilin (Fig. 5B).

Chronophin (CIN, PDXP) is a haloacid dehalogenase phosphatase that also dephosphorylates cofilin. Alteration of CIN activity, through overexpression of either the wild‐type or phosphatase‐inactive mutant CIN, interferes with actin dynamics, cell morphology and cytokinesis 46. In Hela cells, PDXP mediates an ATP‐sensing mechanism for cofilin dephosphorylation. HSP90 binds PDXP, and our results suggest a model whereby attenuated interaction between PDXP and HSP90 during ATP depletion enhances PDXP‐dependent cofilin dephosphorylation and consequent rod assembly. This agrees with the proposed mechanism for the formation of pathological actin/cofilin aggregates during neurodegenerative energy flux 47. Furthermore, Lee *et al*. suggest that in cancer, doxorubicin‐triggered RhoA activation is associated with phospho‐cofilin dephosphorylation due to PDXP activation 48, and LPS was reported to dephosphorylate cofilin *via* PDXP induction 49. These data support our observations on LPS‐induced cofilin dephosphorylation, as depicted in Figure 4. The important role of PDXP on endothelial barrier function is highlighted by the observations that (*i*) silencing PDXP protects HLMVEC against the LPS insult (Fig. 6B), (*ii*) that p53 silencing prevents 17AAG‐induced PDXP suppression (Fig. 6A) and (*iii*) that Nutlin‐induced p53 up‐regulation prevents the LPS‐triggered PDXP induction (Fig. 6C).

We have previously demonstrated that p53 induction can disrupt the inflammatory RhoA signalling. To further elucidate this phenomenon, we investigated the effect of P53 on the regulation of p190RhoGAP, a RhoA‐negative regulator 5, 50. “Super P53” mice express elevated levels of P190RhoGAP (Fig. 5B). Silencing of p190RhoGAP in HLMVEC resulted in a partial protection against LPS‐induced barrier dysfunction (Fig. 7A). Furthermore, LPS was able to strongly suppress the expression levels of p190RhoGAP and AUY922 pre‐treatment opposed that effect (Fig. 7B). The role of p190RhoGAP in endothelial barrier function has been previously established; angiopoietin‐1 attenuation of LPS‐induced endothelial barrier dysfunction *in vitro* and lung oedema *in vivo* was shown to be blocked by p190RhoGAP suppression 51. Induction of P190RhoGAP by P53 may be due to reduced levels of MDM2, an E3 ligase and p53‐negative regulator. MDM2 and P53 are the components of a tightly regulated loop; P53 induction signals MDM2 reduction; and vice versa 52. The downstream effector of RhoA, MLC2, was activated by LPS in both wild‐type and super p53 mice. 17AAG pre‐treatment prevented that effect (Fig. 6C). The effect of LPS in super p53 mice was less robust than in wild‐type mice, likely due to the elevated p53 levels in the super p53 mouse lungs (Fig. 5B).

In conclusion, our study supports and advances our previous observations on the protective role HSP90 inhibitors and P53 on endothelial barrier regulation 4, 12, 53 and provides a new model of the role of this molecule in the mediation of inflammatory responses in the vasculature. The scheme presented in Figure 7D summarizes the hypothesis, based on our findings. The induction of the Rac1 cascade by HSP90 inhibition results in the PAK1‐mediated and p53‐dependant phosphorylation of cofilin. Additionally, P53 suppresses the inflammatory RhoA pathway and the consequent pMLC2 formation by inducing p190RhoA, the RhoA‐negative regulator. Thus, P53 acts as a molecular switch which balances the opposing effects of Rac1 and RhoA in human and murine pulmonary microvascular endothelial cells.

## Conflict of interest

The authors confirm that there are no conflicts of interests.
